# Supplementary material for: Iterative improvement in the automatic modular design of robot swarms
Source: PeerJ Comput Sci. 2020 Dec 7;6:e322. doi: 10.7717/peerj-cs.322 (PMC7924708; doi:10.7717/peerj-cs.322)
Supplement: Supplemental Information 3 [file peerj-cs-06-322-s003.zip › argos3/doc/api/standalone/a00641_source.html]

ARGoS: plugins/simulator/visualizations/qt-opengl/qtopengl\_log\_stream.h Source File


- Main Page
- Related Pages
- Namespaces
- Classes
- Files

- File List
- File Members

# plugins/simulator/visualizations/qt-opengl/qtopengl\_log\_stream.h

Go to the documentation of this file.

```
00001 
00007 #ifndef QTOPENGL_LOG_STREAM_H
00008 #define QTOPENGL_LOG_STREAM_H
00009 
00010 namespace argos {
00011    class CQTOpenGLLogStream;
00012 }
00013 
00014 #include <QTextEdit>
00015 #include <argos3/core/simulator/simulator.h>
00016 #include <argos3/core/simulator/space/space.h>
00017 #include <argos3/core/utility/string_utilities.h>
00018 
00019 namespace argos {
00020 
00021    class CQTOpenGLLogStream : public std::basic_streambuf<char> {
00022 
00023    public:
00024 
00025       CQTOpenGLLogStream(std::ostream& c_stream,
00026                          QTextEdit* c_textedit) :
00027          m_cStream(c_stream),
00028          m_pcTextEdit(c_textedit),
00029          m_cSpace(CSimulator::GetInstance().GetSpace()) {
00030          /* Copy the original stream buffer */
00031          m_pcOldStream = m_cStream.rdbuf();
00032          /* Replace the streambuffer */
00033          m_cStream.rdbuf(this);
00034       }
00035 
00036       virtual ~CQTOpenGLLogStream() {
00037          /* Replace old buffer */
00038          m_cStream.rdbuf(m_pcOldStream);
00039       }
00040 
00041       virtual int_type overflow(int_type t_value) {
00042          if (t_value == '\n') {
00043             std::string strTmp(m_strBuffer);
00044             Replace(strTmp, "<", "&lt;");
00045             Replace(strTmp, ">", "&gt;");
00046             strTmp = "<b>[t=" + ToString(m_cSpace.GetSimulationClock()) + "]</b> " + strTmp;
00047             m_pcTextEdit->append(strTmp.c_str());
00048             m_strBuffer.erase(m_strBuffer.begin(), m_strBuffer.end());
00049          }
00050          else {
00051             m_strBuffer += t_value;
00052          }
00053          return t_value;
00054       }
00055 
00056       virtual std::streamsize xsputn(const char* pc_message,
00057                                      std::streamsize un_size) {
00058          /* Add the message to text stream */
00059          m_strBuffer.append(pc_message, pc_message + un_size);
00060          size_t nPos = 0;
00061          do {
00062             /* Look for a newline */
00063             nPos = m_strBuffer.find('\n');
00064             if (nPos != std::string::npos) {
00065                /* Newline found: display text from the beginning of the stream till the newline */
00066                /* Get the portion to display */
00067                std::string strTmp(m_strBuffer.begin(), m_strBuffer.begin() + nPos);
00068                /* Format the text to display */
00069                Replace(strTmp, "<", "&lt;");
00070                Replace(strTmp, ">", "&gt;");
00071                strTmp = "<b>[t=" + ToString(m_cSpace.GetSimulationClock()) + "]</b> " + strTmp;
00072                /* Append it to the text windoe */
00073                m_pcTextEdit->append(strTmp.c_str());
00074                /* Erase the displayed portion from the text stream */
00075                m_strBuffer.erase(m_strBuffer.begin(), m_strBuffer.begin() + nPos + 1);
00076             }
00077          } while (nPos != std::string::npos);
00078          /* No more newlines found, return */
00079          return un_size;
00080       }
00081 
00082    private:
00083 
00084       std::ostream& m_cStream;
00085       std::streambuf* m_pcOldStream;
00086       std::string m_strBuffer;
00087       QTextEdit* m_pcTextEdit;
00088       CSpace& m_cSpace;
00089    };
00090 
00091 }
00092 
00093 #endif
```

---

Generated on 10 Jul 2018 for ARGoS by 
 1.6.1
